# A Inteligência Artificial Pode Mudar Nossa Interpretação dos Escores de Risco Cardiovascular?

**DOI:** 10.36660/abc.20240280

**Published:** 2024-07-01

**Authors:** Maria Cristina Meira Ferreira, Glaucia Maria Moraes de Oliveira

**Affiliations:** 1 Universidade Federal do Rio de Janeiro Rio de Janeiro RJ Brasil Universidade Federal do Rio de Janeiro, Rio de Janeiro, RJ – Brasil

**Keywords:** Infarto do Miocárdio com Elevação do ST, Síndrome Coronariana Aguda, Inteligência Artificial, Intervenção Coronária Percutânea

A necessidade de buscar meios de predizer a ocorrência de eventos futuros é um fascínio da raça humana desde seus primórdios. Através de observações armazenadas na memória humana, a previsão de eventos fazia parte da segurança e sobrevivência da humanidade. Com o decorrer dos séculos, a necessidade de previsão se tornou parte de diversas ciências pela extrema importância na orientação de tomada de decisões estratégicas, na medicina não foi diferente.

O ser humano desenvolveu habilidades em registrar, organizar e armazenar dados para uma análise futura. Passamos do armazenamento analógico para o digital e, junto ao desenvolvimento dos métodos estatísticos, enormes bancos de dados passaram a ser possível de analise. Na medicina, o emprego de algoritmos complexos pelos sistemas computacionais, passou a prever risco de desenvolvimento de doenças, prognósticos, melhores estratégias terapêuticas, risco de eventos, mortalidade, dentre outros.^1^

Em cardiologia, diversos estudos descrevem maior mortalidade em mulheres por infarto agudo do miocárdio com supra se ST (STEMI), comparada aos homens, sendo as prováveis causas amplamente debatidas.^2^ Maior percentagem de comorbidades no sexo feminino seria o suficiente para justificar?^3^ Diferenças no tratamento ou situações biológicas específicas do sexo feminino poderiam estar envolvidas?^4^ Análise do registro SWEDEHEART discorda desta diferença de mortalidade, afirmando que em uma análise mais precisa, com ajustamento de diversos fatores, a diferença pode não existir. Entre eles dados demográficos, comorbidades, tempo adequado de utilização da melhor terapia, uso prévio de fibrinolíticos, acesso vascular para angioplastia primária e a associação de terapia medicamentosa.^5,6^

Neste estudo,^7^ os autores discorrem sobre esta questão, avaliando a diferença da mortalidade intra-hospitalar entre os sexos dos pacientes atendidos com STEMI em um hospital terciário. Baseado nos resultados, questionam a eficácia do GRACE score em prever mortalidade igualmente em ambos os sexos.

O GRACE score, desenvolvido por um estudo nos primeiros anos deste século, é amplamente utilizado até hoje para predizer mortalidade nas síndromes coronarianas agudas. O escore inclui, entre os fatores preditores, algumas comorbidades e o estado clínico-laboratoriais dos pacientes, mas não inclui sexo.^8^ A medicina tem um dinamismo acentuado em relação as melhores estratégias diagnósticas e terapêuticas, portanto o GRACE score vem sendo questionado, não somente por não incluir sexo, como também outros fatores.^9–11^ Embora um maior número de comorbidade tenha sido considerado uma possível justificativa para maior mortalidade no sexo feminino, algumas comorbidades fazem parte do cálculo do GRACE escore e há evidências na literatura que suportam que o escore possui acurácia igual a ambos os sexos.^12^

Um ponto comum nos diversos estudos sobre doença coronariana se refere ao baixo número de mulheres incluídas nos estudos clínicos.^13^ O artigo de Silva J. et al. reflete essa realidade com inclusão de 36% de pacientes do sexo feminino. Mulheres são a maioria da população e são frequentemente subestudadas, esta discrepância necessita ser corrigida, principalmente se desejamos desenvolver medidas específicas para este sexo. Um outro estudo brasileiro de análise de validação do Grace score, 60% da população foi do sexo masculino.^14^

Porém, chama atenção no estudo de Silva J et al.^7^ a diferença na qualidade de tratamento entre os sexos. O tempo total de isquemia maior nas mulheres é estatisticamente muito significativo (p<0,001), podendo refletir o pior desfecho. Ao analisarmos os diversos subintervalos, considerando desde o início da dor até o momento da reperfusão por balão de angioplastia coronária (*pain-balloon*), nenhum subintervalo identificou diferença estatisticamente significativa entre os sexos que justificasse o atraso na terapêutica de reperfusão do sexo feminino. Desta forma, fica a dúvida qual estágio do atendimento possamos responsabilizar pela terapêutica menos adequada oferecida ao sexo feminino, o que representa um fator fundamental para orientação de prover um tratamento igualitário a ambos os sexos.

O artigo traz um importante tema a discussão, chamando a atenção quanto a necessidade de corrigir falhas para na instituição da melhor terapêutica para as mulheres. Escore de risco fazem parte de uma interligação de sistemas complexos e dinâmicos que interagem diversos fatores entre si, devendo estar em evolução constante. Interconexões limitadas e falta de dinamismo tornam os escores menos preditivos. Assim ocorre com o GRACE score, onde diversos estudos procuram uma maior associação de fatores interrelacionados para melhorar sua precisão prognóstica. A questão da necessidade de incluir sexo, fator único, para melhor precisão prognóstica, permanece em aberto, especialmente a luz dos conhecimentos atuais em que a utilização de algoritmos complexos identificando múltiplas associações entre variáveis aumentam efetivamente a precisão das análises.
