# Supplementary material for: A Inteligência Artificial Pode Mudar Nossa Interpretação dos Escores de Risco Cardiovascular?
Source: Arq Bras Cardiol. 2024 Jul 1;121(4):e20240280. [Article in Portuguese] doi: 10.36660/abc.20240280 (PMC12087633; doi:10.36660/abc.20240280)
Supplement: Translation

# Translation: Can Artificial Intelligence Change our Interpretation of Cardiovascular Risk Scores?

**DOI:** 10.36660/abc.20240280i

**Authors:** Maria Cristina Meira Ferreira, Glaucia Maria Moraes de Oliveira

**Affiliations:** 1 Rio de Janeiro RJ Brazil Universidade Federal do Rio de Janeiro, Rio de Janeiro, RJ – Brazil

**Keywords:** ST Elevation Myocardial Infarction, Acute Coronary Syndrome, Artificial Intelligence, Percutaneous Coronary Intervention

The need to discover ways to predict future events has fascinated the human race since its beginnings. Through observations stored in human memory, predicting events was part of security and survival of humanity. Over the centuries, the need for prediction became part of several sciences, due to its extreme importance in guiding strategic decision-making, and in medicine it wasn't different.

Human beings have developed skills in recording, organizing, and storing data for future analysis. We went from storing in analog mode to digital, and along with the development of statistical methods, analysis of large databases has become possible. In medicine, complex algorithms used by computer systems began to predict the risk of developing diseases, prognoses, the best therapeutic strategies, risk of events, mortality, among others.^1^

In cardiology, several studies have described higher mortality in women with ST-elevation acute myocardial infarction (STEMI), compared to men, and probable causes are worldwide debateted.^2^ Would a higher percentage of comorbidities in females be enough to justify it?^3^ Could differences in treatment or female-specific biological situations be involved?^4^ Analyses of the SWEDEHEART registry disagree with this difference in mortality, stating that in a more precise analysis, with adjustment for several factors, the difference may not exist. Among them are demographic data, comorbidities, adequate time to apply the best therapy, use of previous fibrinolytic therapy, vascular access for the primary angioplasty, and the associated drug therapy.^5,6^

In this study,^7^ the authors discuss this issue by evaluating in-hospital sex mortality differences of patients treated for STEMI in a tertiary hospital. Based on the results, the effectiveness of the GRACE score in predicting mortality equally in both sexes is discussed.

The GRACE score, developed in a study caried out in the first years of the century, is widely used to predict mortality in acute coronary syndromes. The score includes, among predictors, some comorbidities and clinical-laboratory status of patients, but does not include sex.^8^ Medicine is remarkably dynamic concerning the best diagnostic and therapeutic strategies; for this reason, the GRACE score is currently questioned, not only for not including sex, but also for other factors involved.^9–11^ Although the higher number of comorbidities was considered a possible explanation for women's mortality, some of these comorbidities are already included in the GRACE score, and evidence from the literature supports that the score is equally accurate for both sexes.^12^

A common point in the different studies of coronary disease refers to the low number of women included in clinical trials.^13^ The article by Silva et al.^7^ corroborates this, with the inclusion of 36% of female patients. Women make up the majority of the population and are often understudied. This discrepancy needs correction in the clinical studies, especially if we want to address specific measures for this sex. In another Brazilian study analyzing the GRACE score validation, 60% of the population was male.^14^

However, the study by Silva et al.^7^ calls attention to the difference in the quality of treatment between the sexes.^7^ Total ischemic time was significantly longer (p<0.001) in women, which may reflect a worse outcome. When analyzing the several subintervals, considering the period from pain onset to the coronary angioplasty (*pain-balloon*), none showed statistically significant differences between the sexes that would justify the delay in reperfusion therapy in women. Therefore, the question of which stage of care could be responsible for the inadequacy in the treatment offered to women remains unanswered, which represents a fundamental issue in providing equal treatment for both sexes.

The article brings back an important topic to discussion, that is to correct key points at this moment to allow providing the best therapy for women.^13^ Risk scores are part of an interconnection of complex and dynamic systems that interact with different factors and must be constantly evolving. Limited interactions and lack of dynamism make scores less predictable.^1^ GRACE score is currently following this new way; many studies are trying to find out more association of interrelated factors to improve its prognostic accuracy.^9–11^ The question of whether we need to include a single factor as sex, to improve prognostic accuracy remains open, especially in light of current knowledge that applying complex algorithms to identify associations between variables effectively increases the precision of analyses.
